# Correction: Kudo, K. Localization Detection Based on Quantum Dynamics. *Entropy* 2022, *24*, 1085

**DOI:** 10.3390/e24111697

**Published:** 2022-11-21

**Authors:** Kazue Kudo

**Affiliations:** 1 Department of Computer Science, Ochanomizu University, Tokyo 112-8610, Japan; kudo@is.ocha.ac.jp; 2 Graduate School of Information Sciences, Tohoku University, Sendai 980-8579, Japan

In the original publication [1], there was a mistake in Figure 1a,b as published. The entanglement entropy data were wrong. The corrected Figure 1a,b appears below. 

Accordingly, corrections have been made to two paragraphs:

- Section 3.1, the third paragraph:

The disorder strength dependence of the half-chain entanglement entropy shown in Figure 1a,b shows a behavior similar to that of Ref. [49], although the values were different because of the differences in the models. For each *L*, the variance (standard deviation) peaks around *w* ≃ 1–5, where the transition or crossover between the thermal and localized phases occurs.

- Section 3.2, the third paragraph:

The average magnetization is *M_z_* ≃ 0 in the weak-disorder region, indicating thermalization. When the disorder is strong enough, *M_z_* ≃ *L*, which is a signature of the memory effect because *M_z_* = *L* in the initial state. The memory effect is characteristic of the localized phase, which was also observed in Ref. [49]. The variance (standard deviation) of the magnetization peaks at a disorder strength slightly weaker than that of the entanglement entropy. Since magnetization fluctuates with time and can have negative values, *δM_z_* is relatively large in the weak-disorder region. Thus, the variance peak of the magnetization in this situation cannot apply to determining the transition or overlap point.

The author stated that the scientific conclusions are unaffected. This correction was approved by the Academic Editor. The original publication has also been updated.

## Figures and Tables

**Figure 1:**
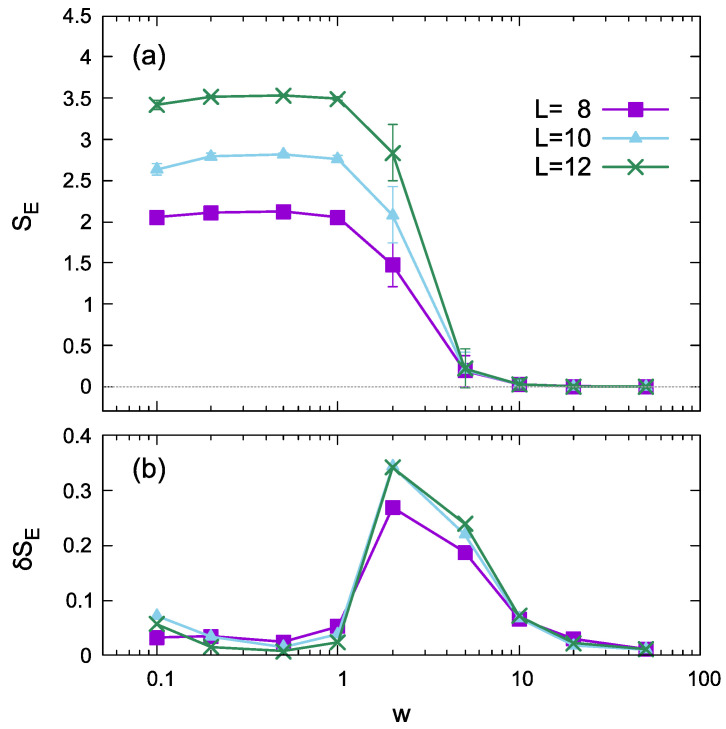
Disorder strength dependence of the entanglement entropy (**a**,**b**) calculated using eigenstates for different system sizes in the noiseless case (σ = 0). (**a**,**b**) plot the half-chain entanglement entropy *S_E_* and its standard deviation *δS_E_* as functions of disorder strength *w*, respectively. The error bars in (**a**) represent standard deviation.
